# Early In Vitro Differentiation of Mouse Definitive Endoderm Is Not Correlated with Progressive Maturation of Nuclear DNA Methylation Patterns

**DOI:** 10.1371/journal.pone.0021861

**Published:** 2011-07-14

**Authors:** Jian Tajbakhsh, Arkadiusz Gertych, W. Samuel Fagg, Seigo Hatada, Jeffrey H. Fair

**Affiliations:** 1 Translational Cytomics Group, Department of Surgery, Cedars-Sinai Medical Center, Los Angeles, California, United States of America; 2 Chromatin Biology Lab, Department of Surgery, Cedars-Sinai Medical Center, Los Angeles, California, United States of America; 3 Bioinformatics, Department of Surgery, Cedars-Sinai Medical Center, Los Angeles, California, United States of America; 4 Liver Disease and Transplantation Center, Department of Surgery, Cedars-Sinai Medical Center, Los Angeles, California, United States of America; 5 Regenerative Medicine Institute, Cedars-Sinai Medical Center, Los Angeles, California, United States of America; University of Insubria, Italy

## Abstract

The genome organization in pluripotent cells undergoing the first steps of differentiation is highly relevant to the reprogramming process in differentiation. Considering this fact, chromatin texture patterns that identify cells at the very early stage of lineage commitment could serve as valuable tools in the selection of optimal cell phenotypes for regenerative medicine applications. Here we report on the first-time use of high-resolution three-dimensional fluorescence imaging and comprehensive topological cell-by-cell analyses with a novel image-cytometrical approach towards the identification of in situ global nuclear DNA methylation patterns in early endodermal differentiation of mouse ES cells (up to day 6), and the correlations of these patterns with a set of putative markers for pluripotency and endodermal commitment, and the epithelial and mesenchymal character of cells. Utilizing this in vitro cell system as a model for assessing the relationship between differentiation and nuclear DNA methylation patterns, we found that differentiating cell populations display an increasing number of cells with a gain in DNA methylation load: first within their euchromatin, then extending into heterochromatic areas of the nucleus, which also results in significant changes of methylcytosine/global DNA codistribution patterns. We were also able to co-visualize and quantify the concomitant stochastic marker expression on a per-cell basis, for which we did not measure any correlation to methylcytosine loads or distribution patterns. We observe that the progression of global DNA methylation is not correlated with the standard transcription factors associated with endodermal development. Further studies are needed to determine whether the progression of global methylation could represent a useful signature of cellular differentiation. This concept of tracking epigenetic progression may prove useful in the selection of cell phenotypes for future regenerative medicine applications.

## Introduction

Pluripotent stem cells such as embryonic stem cells provide an exciting alternative source for hepatocyte lineage cells, to study early liver organogenesis, and in the creation of an unlimited source of donor cells for hepatocyte transplantation therapy of patients with end-stage liver diseases, due to cadaveric organ shortage [1] —which first needs to be explored in mammalian models. Murine embryonic stem (mES) cells have been directed in vitro to produce almost all cell types derived from the definitive endoderm, mesoderm, and ectoderm [2]. Increasing evidence supports the hypothesis that fate decisions in ES cell cultures reflects a series of binary choices between alternate cell states mimicking lineage commitment during developmental processes in the mammalian embryo [3]–[5]. However, much evidence indicates that the pluripotent cell populations in the embryo or in ES cell cultures are not comprised of a single cellular entity, but instead display significant heterogeneity at the molecular level —heterogeneity that is associated with an apparent probabilistic element of fate determination. Apparently the molecular heterogeneity in human ES cultures is reflected by the variability in expression of cell surface antigens seen under culture conditions that promote stem cell renewal. In search of the mechanisms that govern pluripotency and ES cell self-renewal, a growing list of evidence highlights chromatin as a leading factor: the study of chromatin structure, dynamics and organization is also central to the understanding of the maintenance of self-renewal and pluripotency, with ES cells currently serving as a gold standard [6]. Recent studies of the ES cell transcriptome and epigenome have revealed that the pluripotent ES cell is characterized by a high degree of plasticity in chromatin structure [7]. Mammalian genomes are highly organized in the three-dimensional space of the nucleus in interphase [8], [9]. The chromatin of pluripotent stem cells is believed to have unique characteristics, including an open conformation and a hyperdynamic association of chromatin proteins, reflecting the plasticity of the genome in pluripotent cells [10], [11], and likely contributing to the maintenance of pluripotency and self-renewal [12], [13]. Interestingly, major and minor satellite repeats, as well as other repetitive sequences, such as telomeric chromatin, which are normally repressed in differentiated cells, seem to be less condensed and highly transcribed in mouse pluripotent ES cells [14]–[20]. DNA methylation is a key regulator of gene expression programming and genome organization in cellular differentiation [21]–[23], and the establishment of DNA methylation patterns proceeds through defined phases during development [24]–[27]. Given the large dynamic range in 5′-methylcytosine (MeC) load during differentiation and the fact that most MeC is nonuniformly distributed in the human genome [21], image-based assessment of methylation patterns, especially MeC patterns in cell nuclei, may provide a powerful technique to characterize cells during differentiation and in their fate as the underlying molecular processes involve large-scale chromatin reorganization, which is visible by light microscopy [28]–[30]. These studies indicate that chromatin organization is profoundly different in embryonic stem cells than in differentiated cells. The genome organization in pluripotent cells undergoing the first steps of differentiation is highly relevant to the reprogramming process during this phase. Considering this fact, chromatin texture patterns that identify cells at the very early stage of lineage commitment could serve as identification tools in the selection of optimal cell phenotypes for regenerative medicine applications. One might anticipate that these phenotypic signatures may help in identifying artifacts of in vitro differentiation systems, utilized in cell-therapies, which could result in aberrant epigenetic imprints [31]. Though unproven it is conceivable that aberrant epigenetic imprints could result in clinically significant gene dysregulation during the therapeutic application. Recently 3-D quantitative DNA methylation imaging (3D-qDMI) was introduced as a cytomic approach that applies image-analysis algorithms for extraction of fluorescence signals from three-dimensional images of chromatin texture to visualize and measure changes in global DNA methylation (MeC) and related chromatin reorganization in nuclei of thousands of cells in parallel [32]–[34]. This capacity to interrogate a population in a cell-by-cell fashion is a powerful means in the analysis of ES cell populations that represent an intrinsically heterogenic system of individual cells with a high level of spatio-temporal complexity [35], [36]: the regulation of pluripotency maintenance and lineage commitment seem to involve rapid switches between both stochastic and binary signaling events, and fluctuations at a single cell level often lead to profound changes in the structure of cell populations [37]. Here we report on using 3D-qDMI for comprehensive topological analyses towards the identification of global nuclear MeC patterns in early endodermal differentiation of mouse embryonic stem cells, and the correlations of these patterns with the expression of a set of markers for pluripotency and endodermal commitment, as well as for the epithelial and mesenchymal character of cells. The investigations should lead to the discovery of MeC-related chromatin textures that could be used as identifiers of multipotency and lineage commitment.

## Results

The objective of our study was to characterize mouse embryonic stem cells during in vitro early differentiation towards definitive endoderm —from 24 hours post cell seeding (24 hps) up to 144 hours post induction of differentiation (day 6)— regarding their global nuclear DNA methylation patterns. We also explored correlations of these patterns with the cellular expression of biomarkers that have been reported to signify either the status of undifferentiation and pluripotency such as Oct-4 or early stages of endodermal differentiation such as the DNA-binding protein forkhead box A2 (FoxA2) and the transcription factor SRY-related HMG-box (Sox17); as well as the cell-cell adhesion molecule E-cadherin (Cdh1), and the insulin-like growth factor 1 receptor (IGFR) involved in cell transformation, which are indicators of the epithelial and mesenchymal character of cells, respectively. Lineage-specific differentiation was induced by culturing the mES cells in the presence of acidic fibroblast growth factor 1 (aFGF), as previously reported [38]. Subpopulations of the cells deriving from one initial batch were cultured in parallel and fixed at 24 hps (after the cells had attached to the culture dish surface), and subsequently at day 3 and day 6 post initiation of differentiation. The 24 hps cell colonies represented undifferentiated mES cells. For the characterization of cells we delineated the patterns of our targets —methylated cytosine (MeC), global DNA, together with the aforementioned biomarkers— using immunofluorescence and high-resolution confocal microscopy. Although the imaging modalities were kept constant during the entire work, we co-visualized MeC and global DNA with six different combinations of two markers (for each day) by immunofluorescence to rule out any signal biases that may arise from inconsistencies in optical imaging. We then analyzed target patterns utilizing 3D-qDMI, a dedicated algorithm we had developed for the purposes of assessing the nuclear topology of global DNA methylation sites as well as colocalization with other nucleic acid and proteinaceous targets [30], [32], [34]. The analyses entailed two main features: a) the codistribution of MeC and global DNA (visualized by DAPI), and b) the overall expression level of each target measured as respective mean intensities, both of them on a per-cell/nucleus base. For each day and combination of targets we were able to collect multiple stacks of 2D images, each containing 400 to 18,000 cells that reflect various sample sizes for statistical purposes. Each image frame was taken from one to three colonies of ES cells. This approach allowed us to analyze the cell populations at three different levels for each day and target: starting from (i) the sum of all colonies that reflect a significant portion of the entire cell population, over (ii) each cell cluster for itself, down to (iii) the characteristics of each individual cell.

### Codistribution patterns of methylcytosine and global DNA progress in early differentiation

**Figure 1:**
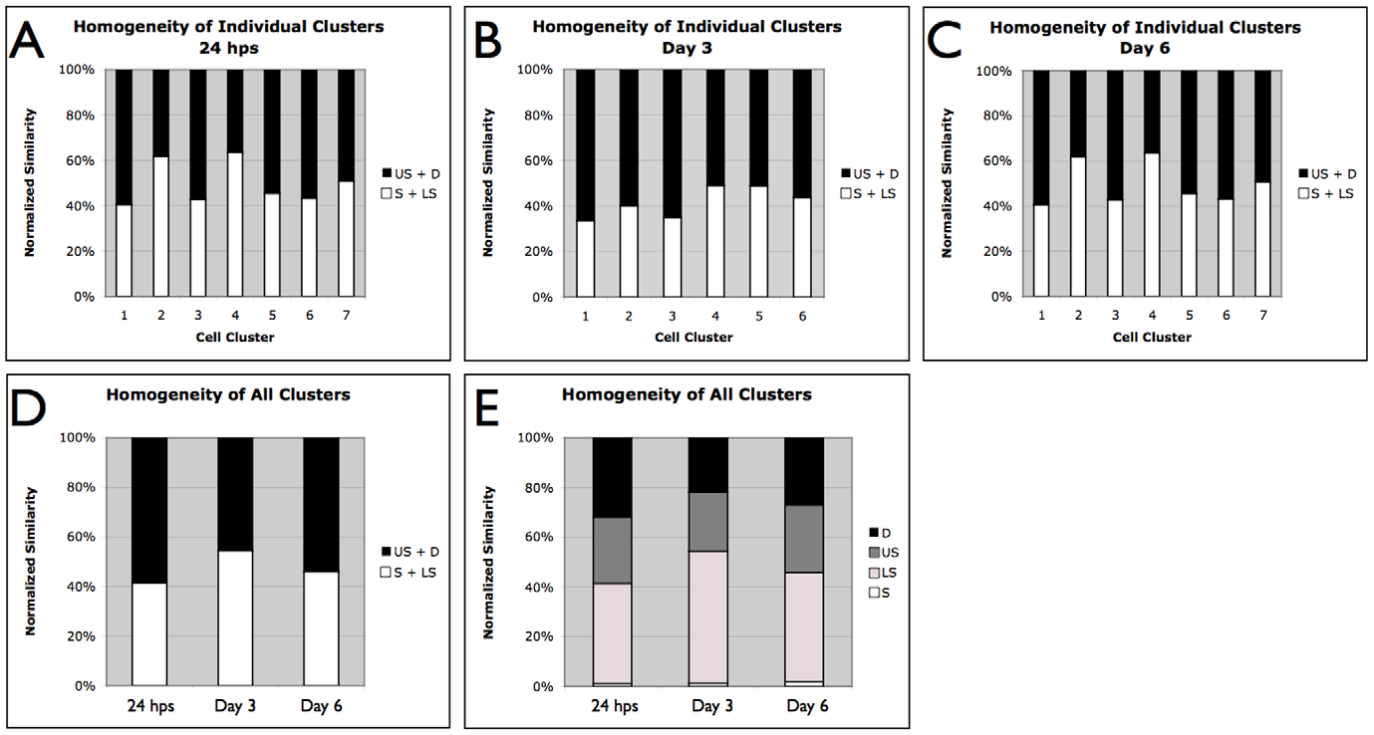
Homogeneity of cell populations regarding MeC/gDNA codistribution patterns. (A–C) The data analyzed for individual cell clusters (n = 7) at 24 hours post seeding (24 hps), and day 3 and day 6 after initiation of differentiation, illustrates the variation in the degree of homogeneity between the different clusters: 25% at 24 hps and 6, and 15% at day 3. This difference is averaged out if all clusters are analyzed together. Herefore, the similar (S) and likely similar (LS) cells were displayed together for representing the total of similar cells (S+LS). Analogously, the counterpart population of dissimilar cells is represented by the sum of unlikely similar (US) and dissimilar (D) cells. (E) In the original split presentation of the four cell categories for the three snapshot days it becomes obvious that the overall dissimilar portion of the imaged populations is continuously comprised of US and D cells in a 50/50 manner, whereas the overall similar populations include a significant majority of LS cells: underlining the high degree of heterogeneity in mES cell populations, apparent even before induced differentiation and persisted during cellular reprogramming.

**Figure 2:**
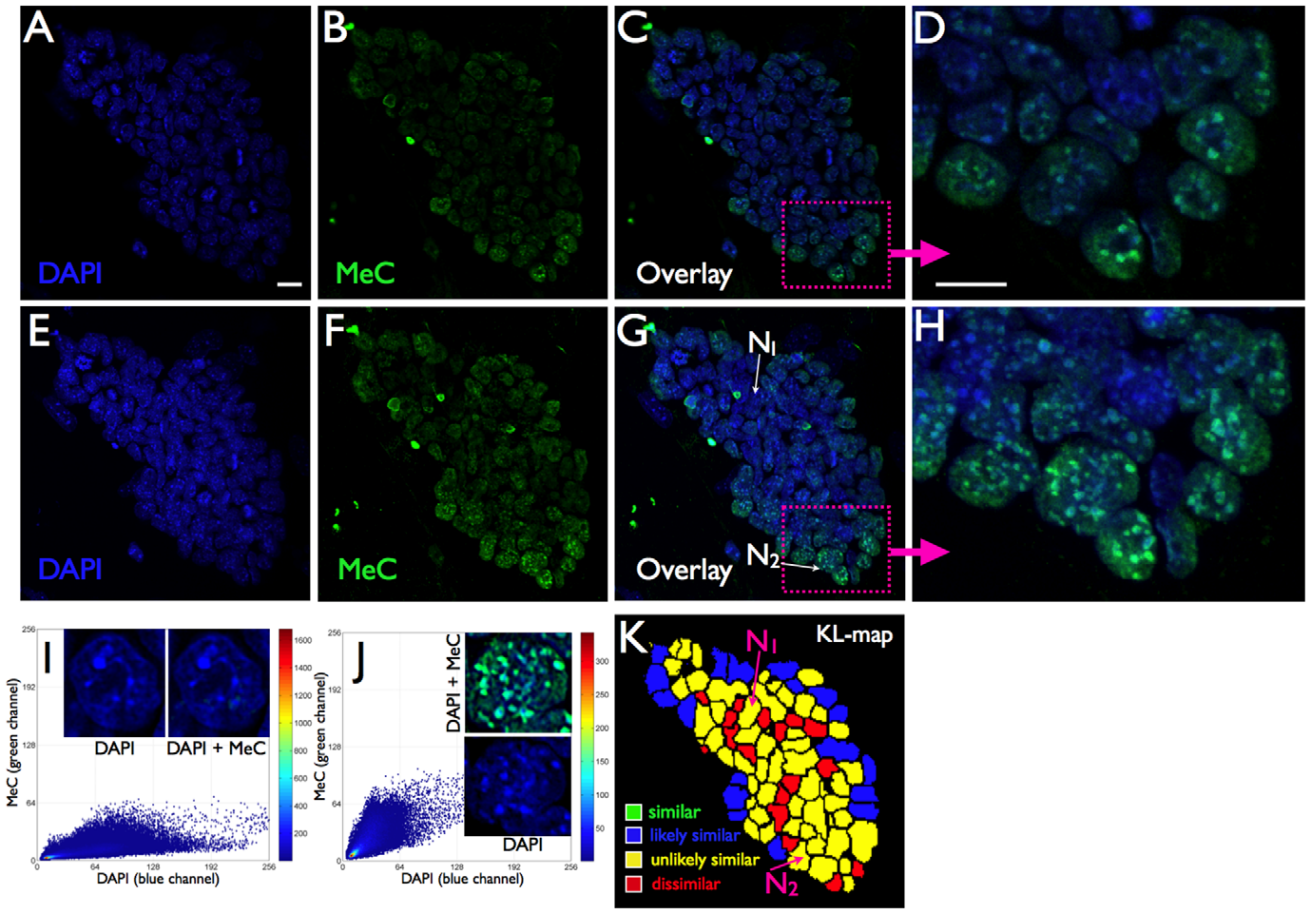
MeC versus global DNA codistribution patterns in mES cells of a sample cluster at 24 hours post seeding and before initiation of differentiation. The upper panel (A–D) represents a confocal mid-section through a 24 hps colony, and the lower panel (E–H) represents the maximum intensity projection of the entire stack of 2D images from the colony for better visualization of the compounded details of fluorescent signals for MeC (green) and global DNA (DAPI-staining = blue) within individual cells. The imaged sample colony, which represents the typical conglomeration of cell subpopulations at 24 hps, consists of a majority of cells that are extremely hypomethylated, with a few cells at the cluster periphery that are partially methylated, as judged by the signal distribution in the overlay images (C and G) and their respective magnified sub-areas (D and H) that detail the typical chromatin sub-structure in mouse cells, in which centromeric and pericentromeric DNA is organized in larger foci termed chromocenters. (I and J) The scatter plots depicting the MeC/DAPI three-dimensional codistribution patterns in representative nuclei N_1_ (almost no global methylation) and N_2_ show significantly different levels of nuclear DNA methylation and reveal that some cells with increased MeC signals may have already undergone stages of spontaneous early differentiation. (K) The KL-map of the cluster (generated from a mid-section of the 2D image stack), which displays the similarity of cells regarding their MeC/DAPI patterns among themselves, further illustrates that the cluster has a high degree of heterogeneity. Scale bars are 10 µm.

**Figure 3:**
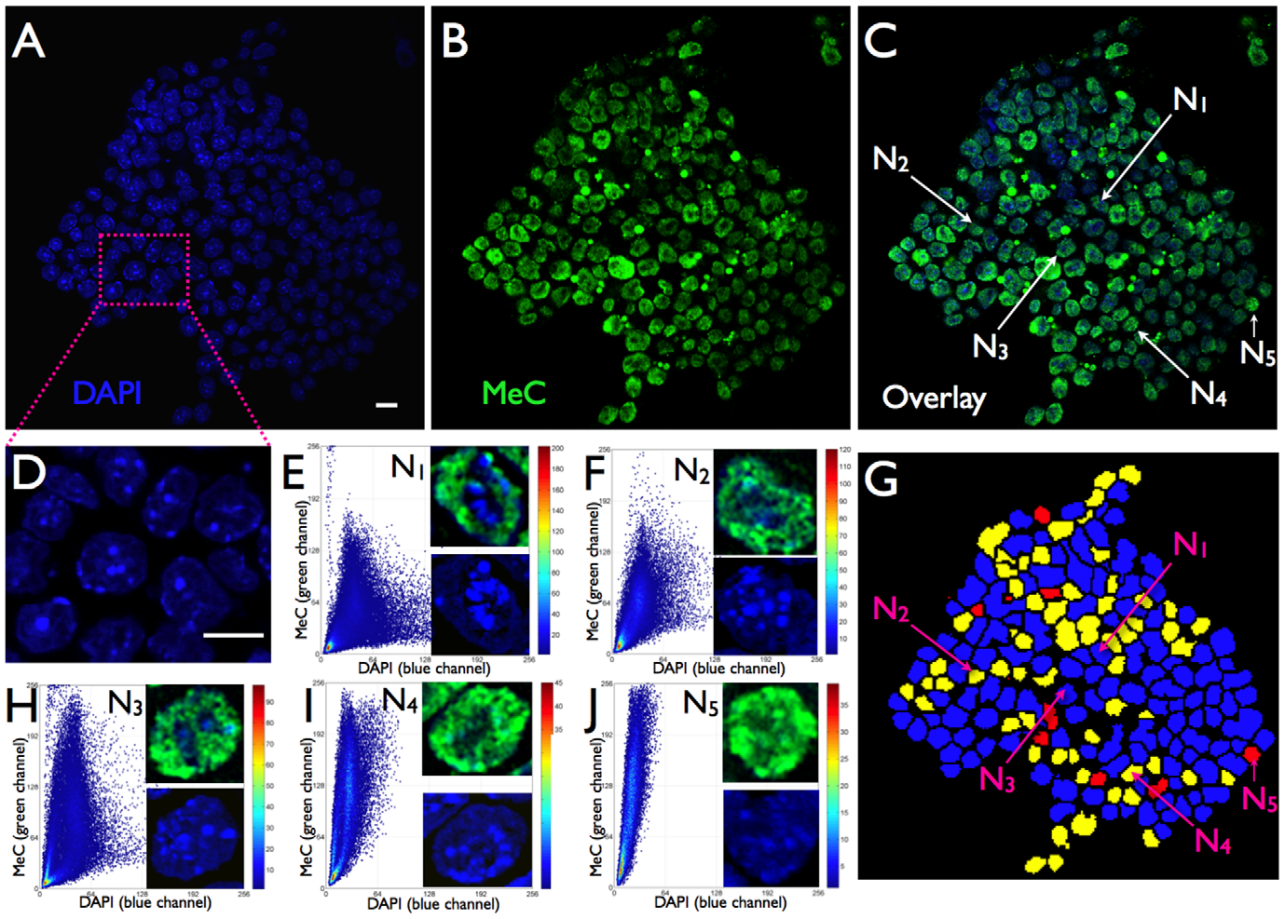
MeC versus global DNA codistribution patterns in mES cells of a sample cluster at day 3. (A–C) A confocal mid-section of a day 3-cell cluster has typically retained its clonal character, meaning that it presents a closed structure with all cells attached to one another. The overall picture has changed compared to the 24 hps-cluster in Figure 2: the day 3 cluster consists of only a few strongly hypomethylated nuclei and a significantly larger number of methylated nuclei (MeC = green). The small bright green speckles represent disintegrated cells (blebs) that have most probably undergone necrosis or apoptosis. (D) The magnification illustrates the uneven distribution of the mouse genome (global DNA) in the nuclei with DAPI-intense chromocenters and less DAPI-positive euchromatic areas. (E, F, H–J) The five selected nuclei (N_1_–N_5_) represent different degrees of global methylation and similarity categories —as detailed in the respective magnified single-channel DAPI and overlay MeC/DAPI images— which contribute to the cluster's heterogeneity. N_1_, N_2_, and N_3_, whose types are more abundant within the cluster, display increasing degrees of euchromatin methylation, and the less frequent N_4_ and N_5_-type of cells also heterochromatin (chromocenters and DAPI-intense regions at the nuclear border) methylation. N5 seems to be nearly completely hypermethylated and embodies the extreme opposite to the rare but almost entirely hypomethylated nuclei within the same cluster. (G) Despite numerous *unlikely similar* cells (yellow), this cluster shows a higher degree of MeC/DAPI pattern homogeneity among its cells —relatively high percentage of *likely similar* cells (blue). Scale bars are 10 µm.

**Figure 4:**
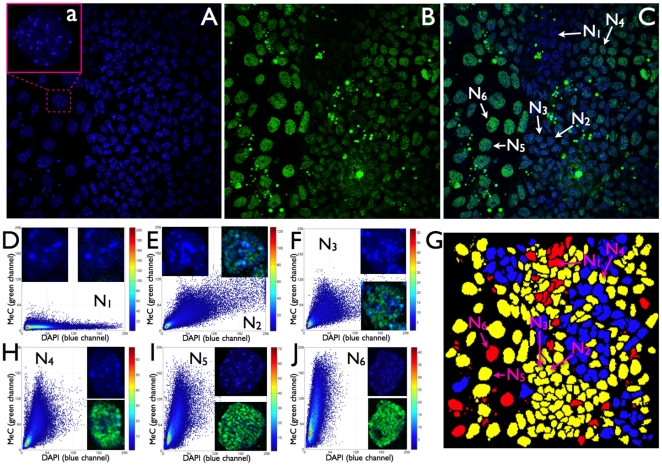
MeC versus global DNA codistribution patterns in mES cells of a sample cluster at day 6. (A–C) A confocal mid-section of a typical day 6-colony represents a more open gestalt, with the stem core of still strongly attached cells with smaller nuclei that show either extreme hypomethylation or slight global DNA methylation (green), and cells with larger nuclei (magnified in subfigure a) that seem to have become detached into the cluster periphery. (D–F, H–J) Selected nuclei of the different categories and degrees of global DNA methylation across the cluster, in which almost entirely methylated nuclei in the transition area —such as N_5_ and N_6_— expose a larger morphology than other cells, which are extremely undermethylated (N_1_) or display intermediate levels of more euchromatic DNA methylation (N_2_, N_3_, and N_4_) as seen in the magnified images of the respective single-channel DAPI and overlay MeC/DAPI images of the nuclei. These latter types of cells are located more in the cluster interior. (G) The KL-map describes the extreme cluster heterogeneity in MeC/DAPI codistribution patterns. Interestingly, the cluster core shows a pocket-like conglomeration of cells of one type of similarity category, whereas the periphery is more mixed in this regard.

We analyzed the codistribution of MeC versus global DNA (DAPI) for each imaged nucleus within cell populations of 24 hps, and days 3 and 6, utilizing this feature as a potential biomarker for the characterization of the cells. For evaluation purposes we assessed the populations and individual cells utilizing KL-divergence measurement [39] that we had successfully applied towards other MeC-pattern analyses in the past [32], [33], and categorized the cells by four ranges of KL-values as: *similar* (S), *likely similar* (LS), *unlikely similar* (US), and *dissimilar* (D). Our analyses revealed a mixture of cells with different types of codistribution patterns, already at 24 hps and continuing through days 3 and 6, thus representing heterogeneity within the populations at all times. When pooling the data for each day the results show a slight decrease in heterogeneity at day 3 (55% S+LS cells) compared to 24 hps (40% S+LS), which then had slightly increased back (to 45% S+LS) on day 6 (Figure 1). When dividing the day-based data into sets, one for each individual cell cluster, we observed a variation in homogeneity between the colonies (n = 7), i.e the portions of S+LS and US+D within the populations varied: between 40 and 65% S+LS cells at 24 hps and day 6, and 35–50% at day 3. However, since only two out of seven colonies showed significantly less heterogeneity (65% S+LS) this variation did not have a strong gravity to shift the pooled data (from all cell clusters). The degree of cell population heterogeneity becomes also obvious at the image level. A sample of a typical 24 hps-cluster displayed in Figure 2 demonstrates that although the majority of cells —especially in the interior of the cluster core— are hypomethylated, the cells show different degrees of global MeC gain. A few cells in the cluster periphery seem to have further advanced in their methylation load and distribution. The nuclei N_1_ and N_2_ and their respective MeC/DAPI codistribution scatter plots represent the two extreme categories of cells within the colony. On day 3 we noticed a strong increase in the number of nuclei within the colonies that have become more methylated. The sample cluster in Figure 3 confirms the overall impression that the individual colonies are slightly reduced in their heterogeneity, but they are still comprised of cells with a variety of different MeC loads and spatial distributions. Furthermore, the colonies still harbor extremely hypomethylated nuclei predominantly located in the cluster core; at the same time the colonies are populated by a majority of nuclei with increased MeC with various degrees of hypermethylation in their euchromatic and heterochromatic regions as presented by the nuclei N_1_ to N_5_: with N_1_–N_3_ showing gradual increase in euchromatin methylation but no significant heterochromatin methylation, and N_4_ and N_5_ displaying a seemingly complete hypermethylation of their entire genomes. This picture is even more prevalent in day 6 colonies as demonstrated in Figure 4. These clusters regularly display a different gestalt than 24 hps and day 3 colonies. The originally closed cluster integrity has vanished and a transition area into a monolayer of detached cells with larger gaps between themselves has become apparent at the cluster periphery. The core is still harbored by hypo-to-less methylated cells, whereas the seemingly detached cells, —which also have a larger nuclear morphology— are more methylated to various degrees with an increased number of extremely hypermethylated cells.

**Figure 5:**
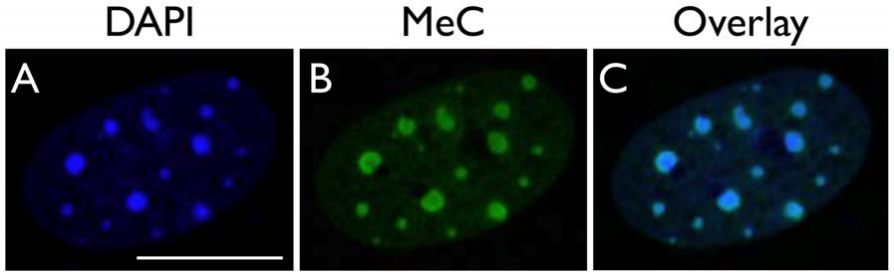
Mouse embryonic fibroblast (MEF). (A) The typical phenotype of a terminally differentiated mouse nucleus such as of a MEF, in which the constitutive heterochromatin of centromeric and pericentromeric DNA aggregates into smaller and larger chromocenters that stand out as bright DAPI-intense foci against the rest of the nuclear genome regions, which harbors less DAPI-intense euchromatin. (B) The chromocenters are heavily methylated and show a similar striking appearance (also in the DAPI pattern), when fluorescently labeled with a specific antibody against 5-MeC. (C) The overlay indicates a strong colocalization of the two types of DNA, especially in the heterochromatic areas. Scale bar is 10 µm.

**Figure 6:**
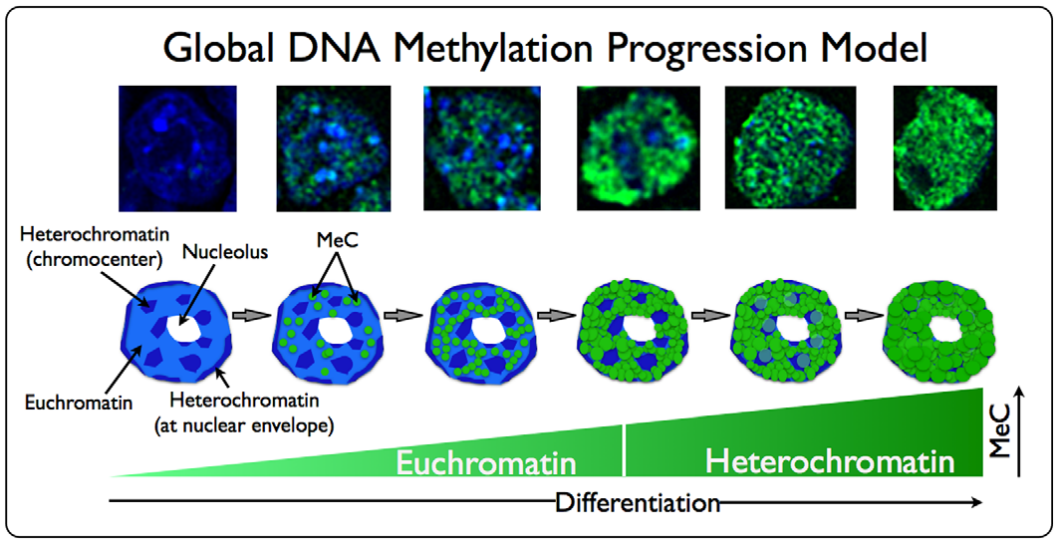
Scheme of the global DNA methylation progression model. (A) The hypothetical model proposes the progression of global DNA methylation of the nuclear genome, reflecting the differential increase of the nuclear MeC load during early differentiation of mouse ES cells: initially extremely hypomethylated cells (located more in the colony interior) become first methylated within their euchromatic parts of their genome that gradually reaches a hypermethylation status, before the heterochromatic regions also become successively methylated, starting with the facultative heterochromatin at the nuclear envelope and then progressing into the constitutive heterochromatin that is typically organized in chromocenters within the nucleoplasm of mouse cells.

In summary, the colony-based analysis of the three day-points revealed that as the populations progress through culturing and cell divisions, more and more cells seem to become differentiated and this concurs with an increase in nuclei at the cluster periphery that are increased in their MeC load compared to the majority of nuclei at 24 hps and the hypomethylated nuclei in the cluster core. Along with an increase of MeC there is also a change in the codistribution of MeC and global DNA to be observed. The imaged populations indicate a MeC/gDNA pattern progression, as judged by an increase in pattern frequency towards nuclear genome hypermethylation from 24 hours post cell seeding to day 6 of initiated differentiation: 24 hps, the phenotype of the majority of the cells that are located in the interior core of the clusters display a high degree of hypomethylation of the entire nucleus, including heterochromatic regions —DAPI-intensive areas at the nuclear border and chromocenters located in the nuclear interior— and euchromatic areas that can be identified as less DAPI-intensive areas. Some cells in the cluster periphery, however, show an increase in the methylation load of their euchromatic regions. We observed that the number of cells with this phenotype has gradually increased in day 3 clusters, and therefore assume that this phenotype along with an increase in MeC is presented by cells that had proceeded towards differentiation. On day 3 another cell phenotype appears, although at a lower frequency than the latter described, in which nuclei show a stronger increase in euchromatin methylation —the euchromatin MeC signal areas grow denser and the MeC-signal intensifies, whereas the majority of heterochromatic areas, specifically the chromocenters stay remarkably hypomethylated. This phenotype resembles the exact reciprocal high-MeC signal distribution in terminally differentiated cells such as mouse embryonic fibroblasts (MEF): with highly methylated chromocenters (brightly-MeC-stained areas) and a comparatively lower methylation of the rest of the nucleus (fainter areas), as shown in Figure 5. The inverse phenotype is found at a higher frequency in day 6 populations, along with a fourth phenotype, in which in addition to euchromatic regions also heterochromatic areas are found increasingly hypermethylated. From the frequency of global MeC phenotypes we assume that during differentiation first the euchromatin and then the heterochromatin becomes hypermethylated. Figure 6 displays our hypothetical global DNA methylation progression model in early mES cell differentiation together with a recollection of fluorescent images of respective sample nuclei. Our results are in line with previous observations made by other investigators that conducted molecular as well as fluorescence imaging analysis [13], [24]–[27].

### Expression of nuclear biomarkers does not correlate with MeC patterns

**Figure 7:**
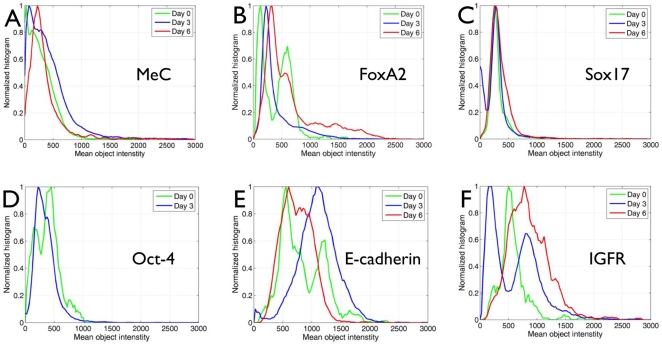
Distribution of biomarkers during differentiation. (A) Initially there coexist two groups of cells with different methylation loads (MeC) —including a large subpopulation with almost no DNA methylation— that converge to one population (Gaussian distribution) with increased maximum MeC level. (B) FoxA2 expression levels also reveal a similar population shift with increasing maximum. (C) The relative distribution of Sox17-positive nuclei as well as the maximum level of its expression does not change in imaged populations over the time 6-day period. (D) Oct-4, initially exhibited two subpopulations also converge to one population by day 3 with a reduced maximum. (E and F) E-cadherin and IGFR expression cells also converge to one group with a large variation. However, whereas the maximum of IGFR is significantly increased over the 6 day period, the maximum of E-cadherin is first increased at day 3 and then reverts to the 24 hps-value, a possible sign for epithelial-to-mesenchymal transition of a large number of cells within the cell clusters.

**Figure 8:**
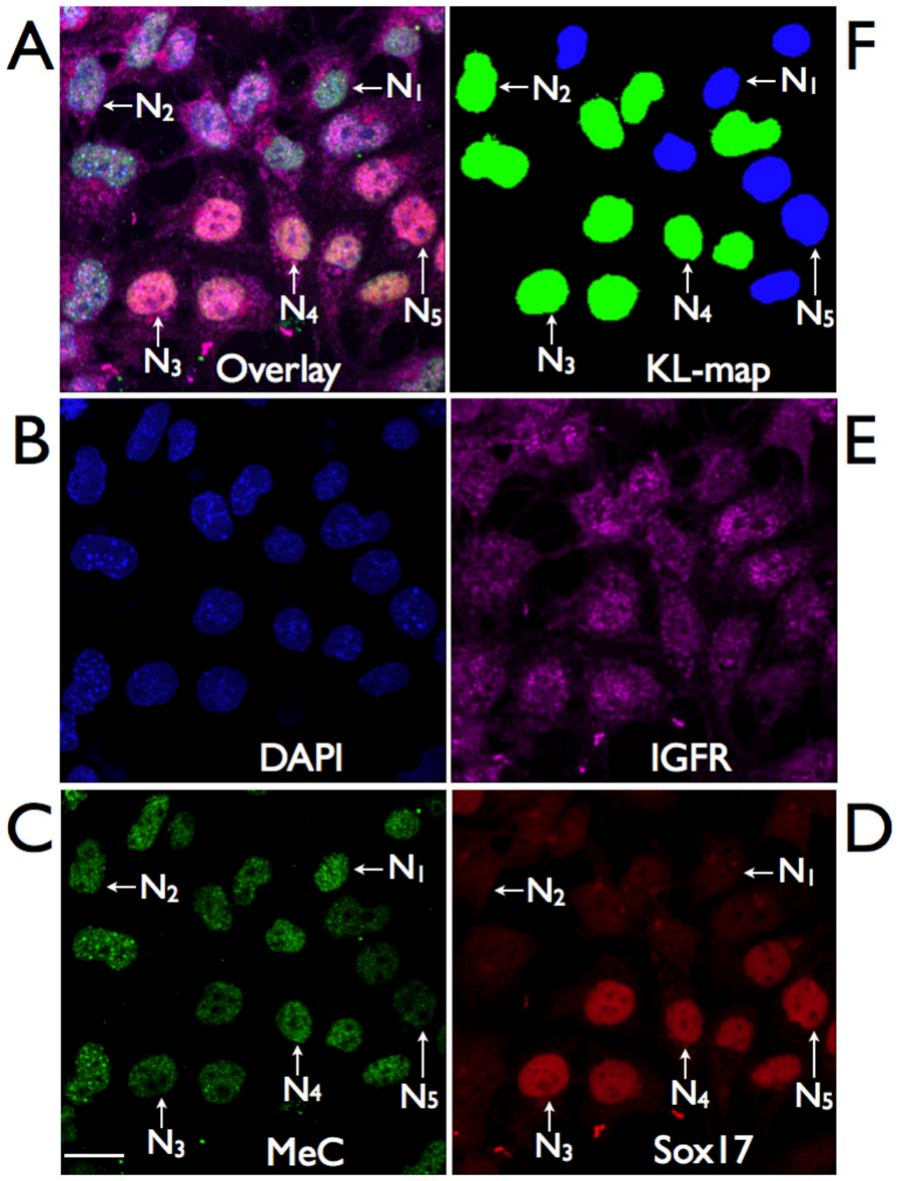
Immunofluorescencent covisualization of MeC and global DNA together with cell-specific markers. (A) The sample four-color image (maximum intensity projection) taken from the peripheral transition area of a day-6 colony delineates the overlay distribution of global DNA (B, blue), methylcytosine (C, green), the endodermal-lineage marker Sox17 (D, red), and the mesenchymal marker IGFR (E, purple). (F) The KL-map indicates a fairly homogeneous subpopulation. The selected nuclei N_1_–N_5_ are similar in MeC/DAPI codistribution but show differential properties regarding their expression levels of the two proteins, specifically Sox17 (described in Fig. 5). The cell population consists of cells that are hypermethylated (green), predominantly in their euchromatic nuclear regions.

In addition to the MeC level of cells, we also measured the abundance of five markers that were covisualized with MeC in multiple combinations, four of them having a nuclear localization. Our notion was to assess (i) any trend in the expression of the markers during differentiation, and (ii) possible correlations among the markers, especially between the MeC loads and/or MeC/DAPI codistribution patterns and marker expression levels. Our visual inspection of the imaged colonies was supported by the abovementioned cytometrical analysis of MeC patterns. The results provide the impression that initially two groups of cells with different DNA methylation loads coexist —including a large subpopulation with almost no DNA methylation— that converge to one population with Gaussian distribution of MeC levels and a slightly increased (∼17%) maximum: the number of cells with extremely high global nuclear MeC intensity has rapidly increased (34% of all analyzed cells) upon induced differentiation by day 3, and has grown even larger (53%) at day 6 (Figure 7). However, the maximum peak shift for global nuclear MeC indicates that individual cells (up to ∼10%) can reach the status of strong methylation already at 24 hps, as spontaneous differentiation of cells may not be totally suppressible. FoxA2 expression levels also reveal a similar population shift with increasing maxima. In comparison, the relative distribution of Sox17-positive nuclei as well as the maximum level of its expression does not change in imaged populations over the 6-day period. This marker shows a much narrower intensity bandwidth than FoxA2. Oct-4 expression is reduced by day 3, indicating that a majority of the cells is most likely loosing their pluripotency. E-cadherin and IGFR are the two markers that show the highest distribution spectrum at all times, with significant changes during differentiation: the IGFR maximum is gradually increased up to ten-fold in the majority of cells, whereas the E-cadherin levels first show an immense (ten-fold) increase on day 3, which on day 6 is reverted to the at 24 hps-level. The latter results are a possible sign for an epithelial-to-mesenchymal transition of a large number of cells within the cell colonies. The data is concordant with the observation that day-6 colonies display a transition area of hypermethylated nuclei with highly elevated levels of IGFR (Figure 8). All markers have a large distribution —equivalent to a large standard deviation— underlining their high heterogeneity in abundance (expression levels) within the cell populations, with the exception of Sox17. Images confirm that this transcription factor is expressed in cells either at higher levels or at the base level (see Figure 8 for example), therefore representing a differentiation marker with a sharper on/off transition. In comparison FoxA2 appears in more blends and thus may exemplify a smoother differentiation marker.

**Figure 9:**
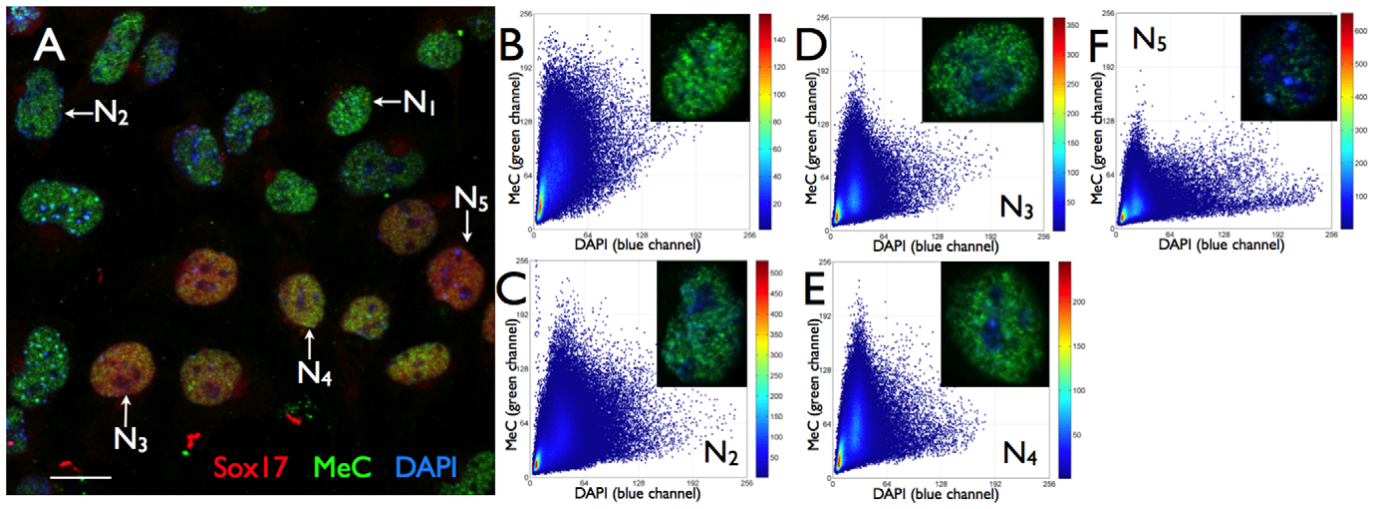
Correlation of MeC and cell-specific markers. (A) A three-color presentation (maximum intensity projection) of the day 6-cell population in Figure 8. (B) The cells' MeC (green) versus DAPI (blue) codistribution patterns are fairly similar as confirmed by the scatter plots of four of the five representative nuclei N_1_–N_4_ (B–E) and the KL-map of the population (F). The cells, however, display different expression levels of the nuclear endodermal-lineage marker Sox17 (red): N_1_ and N_2_ present only background levels, whereas N_3_ and N_4_ show extremely strong signals almost evenly distributed over the nuclear regions that are devoid of nucleoli (also see subfigures C and D for comparison). N_5_ on the hand is less methylated than the other four selected nuclei, but shows high Sox17 expression. Thus, the sample image demonstrates that there seems to be no significant correlation between MeC load/patterns and marker expression in the heterogeneous cell sample.

Furthermore, we did not find any significant correlation between MeC and any of the five markers, in the entire day-based data set (sum of all cell colonies for each day) as well as for each individual cluster. However, a few individual colonies showed a low (0.58) or high (0.86) correlation between MeC and Sox17 at 24 hours after cell seeding, but not at the other days (data not shown). Figure 8 displays a typical sample subpopulation of nuclei at day 6 that is located in the peripheral transition area of its originating cell cluster. The cells were labeled for Sox17 and IGFR in addition to MeC and DAPI-staining. The sample shows that cells can have similar MeC loads and MeC/DAPI codistribution patterns but represent very different Sox17 expression levels from basal background to highest levels, as detailed for selected nuclei N_1_–N_4_ in Figure 9. On the other hand cells that are comparatively less methylated, such as N_5_, display a relatively high Sox17 expression. Table 1 resumes the lack of correlation between the two most relevant endodermal lineage markers, FoxA2 and Sox17, and the methylcytosine load in cell nuclei at all three days, which also results in a lack of correlation between the cells' MeC/DAPI patterns and the markers' nuclear abundance. On the contrary, some of the markers showed an increasing correlation among themselves from 24 hps (0.39–0.65) towards day 3 (0.70–0.85), which had not changed much at day 6. The highest correlation calculated in a colony was found to be between E-cadherin and IGFR (0.96). We experienced a variation of the degree of correlations between the same markers in different cell colonies, even of the same cell population. Based on this result, when pooling the data for all imaged colonies representing a large subset of the overall cultured cell population, we found that this correlation significantly decreased. Our interpretation of these facts is that there is a large variance in colony composition of cells at early lineage differentiation between 24 hours post seeding and day 6 after induction of differentiation, and that eventually each cluster represents its specific cell diversity. Therefore each cell cluster needs to be analyzed separately.

**Table 1:** Correlation between methylcytosine and endodermal lineage markers.

	MarkerProtein	Total Cells(100%)	R*(MeC/Protein)	High-ExpressorCells	R(MeC/Protein)	Highest-ExpressorCells	R(MeC/Protein)
**24hps**	FoxA2	429	0.10	55(12.8%)	0.27	10(2.3%)	0.19
**24hps**	Sox17	1306	0.19	162(12.4%)	0.01	58(4.4%)	0.07
**Day3**	FoxA2	7978	0.09	1174(14.7%)	0.03	439(5.5%)	0.05
**Day3**	Sox17	16430	0.06	1239(7.5%)	0.12	568(3.5%)	0.12
**Day6**	FoxA2	4269	0.03	717(16.8%)	0.03	288(6.7%)	0.08
**Day6**	Sox17	3909	0.15	301(7.7%)	0.09	113(2.9%)	0.23

*R = Pearson's correlation between the mean nuclear intensities of MeC and protein marker.

No significant correlation between the nuclear abundance of the two protein markers, FoxA2 and Sox17, and the MeC load was measured for mES cells 24 hours post seeding (24 hps)/before induction of differentiation and during the first six days in the early development of cells. The analysis was performed for three sets of data for each of the three sampling days: including all cells co-stained for the three targets (total cells), and smaller subsets of these cells that display an overall marker expression >1-fold and >2-fold standard deviation above the average nuclear intensity (high-expressor cells and highest-expressor cells, respectively).

**Figure 10:**
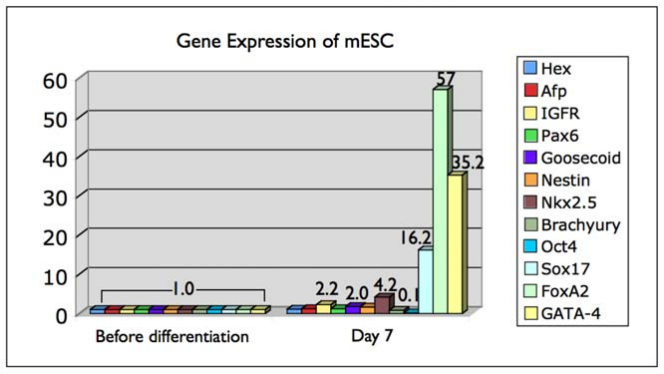
Comparative quantitative RT-PCR of mESC cells. 12 genes relevant to pluripotency and endodermal lineage commitment were examined regarding their differential expression level in cells at day 7 relative to undifferentiated embryonic stem cells (24 hps) before induction of differentiation (1.0 baseline); with ACTB used as an internal control for normalization. A slight increase in mRNA levels was measured for IGFR (3-fold), Goosecoid (2-fold) Nkx2.5 (∼4-fold), and a significantly higher increase for Sox17 (16-fold) GATA-4 (35-fold) and FoxA2 (57-fold). In comparison, Oct-4 was reduced by a factor of 10 towards day 6.

Additionally, we performed quantitative real-time polymerase chain reaction (qRT-PCR) for a set of 12 genes relevant to pluripotency and endodermal lineage commitment, including hex, afp, IGFR, Pax6, Goosecoid, nestin, Nkx2.5, Brachyury, Oct-4, Sox17, FoxA2, and GATA-4, with mRNA collected from the entire cultured cells. Absolute levels of mRNA were normalized to the expression level of the β-actin gene (ACTB). We observed a differential expression of a subset of these markers in cells at day 7 relative to undifferentiated embryonic stem cells (24 hps and before induction of differentiation): a two to three-fold moderate upregulation for IGFR and Goosecoid, a stronger transcript increase of nearly four-fold for Nkx2.5, and a remarkably strong upregulation of Sox17 (16-fold), GATA-4 (35-fold), and FoxA2 (57-fold); on the other hand Oct-4 was reduced by a factor 10 at day 7 (Figure 10). The results are in line with the immunofluorescence data in regards of expression-level trends for the five commonly analyzed markers. Especially, both methods confirm the increase in overall expression for Sox17 and FoxA2 during differentiation.

## Discussion

The notion of our research was to characterize mouse embryonic stem cells during early in vitro differentiation towards definitive endoderm as a model for accessing the relationship between differentiation and global nuclear DNA methylation patterns. Specifically, we explored the correlations of these patterns with the cellular expression of the pluripotency marker Oct-4, the endodermal differentiation markers FoxA2 and Sox17 as well as the epithelial marker E-cadherin and the ubiquitous cell survival marker IGFR. The cell-by-cell analysis through quantitative imaging of DNA methylation and protein expression allowed us to assess cultured embryonic cells in three different ways: 1) the total of all imaged cells as a statistically significant portion of all cultured cells, 2) individual cell colonies as distinct from one another, and 3) single cells selected and compared within one colony or across multiple clusters. The post-imaging combination of data allows for the assessment of possible correlations between cell phenotypic markers. More concretely, ES cells that form small subpopulation or clusters may be more appropriately analyzed in that unit. Compared to the majority of cells before induction of growth-factor mediated differentiation, we observed an increased number of cells with a gain in global DNA methylation towards day 6. The different types of DNA methylation phenotypes occur on average in a marginal portion of cells in 24 hps-colonies —possibly due to spontaneous differentiation— and expand to a majority of cells on day 3 and 6 with an increasing number of extremely hypermethylated nuclei (∼34% and ∼53%, respectively). This tendency indicates a progression of global DNA methylation. We postulate that this progression entails a differential increase in DNA methylation as judged by the MeC/gDNA codistribution patterns we obtained from the analysis of all cells within the imaged colonies: first euchromatic genome regions become gradually hypomethylated before heterochromatic areas follow until almost the entire nuclear genome seems to become hypermethylated. The observations are concordant with the distribution analysis for the MeC load measured as the overall mean intensity of each nucleus. We observed that the maximum load value does not change much between the days, however at the same time the relative number of nuclei with maximum MeC load increases over the differentiation period. The existence of extremely hypomethylated cells in day-3 and day-6 colonies could be reasoned two-fold: (i) some embryonic stem cells at very early stages of lineage commitment may divide asymmetrically and give rise to undifferentiated cells that are extremely hypomethylated, or (ii) conceivable autocrine/paracrine signalling keeps some cells in their undifferentiated status. Furthermore, it has been reported that mitotic inheritance of genome-wide methylation profiles is less stable in ES cells than in somatic cells, and that this epigenetic instability is likely to introduce unpredictable phenotypic variation into clonal populations of ES cells [40]–[42]. We wonder, whether this phenomenon explains the observations in our cell populations. However, these fluctuations do not seem to affect the higher MeC-relevant organization of the genome and its progression during differentiation. Our results concur with related observations, which all indicate a strong hypomethylation of pluripotent cells before lineage commitment and a rapid accumulation of genome-wide methylation during differentiation [14]–[20], [24]–[26], [43]. However, these studies did not track the relationship between lineage-associated biomarkers and the progression of methylation.

In this investigation the aforementioned biomarkers that were covisualized with MeC and global DNA (DAPI) yielded the finding that we could not detect any significant correlation between biomarker expression and the degree of global DNA methylation. This was true, whether assessing the pooled data or in comparison of individual clusters. Even the examination of individual cells with either lowest or highest marker values did not point to any correlation between either marker with MeC. We assume that this variance in marker expression is eventually due to stochastic activity of transcriptional networks associated with endodermal differentiation, a situation referred to as the probabilistic nature of early differentiation. The only high correlation between IGFR and E-cadherin is most plausibly due to the exclusive nature of cells to be either epithelial-like or more of a mesenchymal type. The peak level in E-cadherin on day 3 could be reasoned with a transition that may occur in a large number of cells from epithelial to mesenchymal phenotypes, an event well described during the migration of endoderm within the primitive streak to become definitive endoderm [44]. The cells seem to later revert back to the epithelial phenotype, again also relevant to either the cells' stochastic nature or the initial detachment of cells from the colonies before they become more differentiated as single cells away from their original colony. We are tempted to speculate that the low correlation between the MeC load and distribution and the endodermal markers FoxA2 and Sox17 may be due to two facts that further need to be assessed. 1) It is open as to whether the time period of 6 days is enough for drawing ultimate conclusions regarding any correlations, that may occur further downstream in a more stable differentiation of the cells. Along the same lines, it would be necessary to evaluate, whether MeC load increases first or the cells become FoxA2 and/or Sox17-positive prior to significant changes in global nuclear DNA methylation, and whether there exists any convergence between the MeC features and any of the markers beyond the 6-day time period. For that, live cell imaging with different fluorophore-expressing reporter constructs are viable tools in addressing these questions. 2) Also, the multiplexed labelling of FoxA2/Sox17 with other lineage-specific markers could possibly lead to the further characterization of embryonic stem cell clusters that may be composed of a heterogeneous gemisch of cells with diverse lineage-specific capacities.

Finally, the comparison of qRT-PCR data —that derived from a large group of sorted cells— and imaging data confirms that in situ cell-by-cell analysis is a valuable method to follow up on the gene expression levels beyond transcription for the following facts. We experienced a non-linear correlation between Sox17 and FoxA2 mRNA expression and protein abundance: on day 7 the respective transcription levels of these two factors was 16-fold and 57-fold higher compared to before initiating differentiation (Fig. 10), whereas the highest relative nuclear protein levels did not change in the case of Sox17, and only increased ∼four-fold for FoxA2 towards day 6 (Fig. 7). We do not assume that the one-day difference in data collection could be the reason for this larger discrepancy. Rather, high-resolution fluorescence imaging provides a more detailed and accurate picture of gene expression on the more final protein level that is single cell-specific. This becomes especially evident for the two endodermal markers, for which qRT-PCR only delivers a compounding average expression value across all cells within a population, whereas the 3D-qDMI data shows that on day 3 and day 6 the imaged cell populations are comprised of cells with a heterogeneous expression level of the two proteins: we could observe a general trend highlighted by an increased number of cells in the populations that express very high levels of the two proteins. However, we could also observe coexisting cells with very low to moderate expression levels of these markers. This fact underlines the power of cytomic approaches to track the individual variability of cells [45], which is important in the characterization of inhomogeneous populations such as ES cell derivatives, and which may become disguised when cells are crudely analyzed. These phenomena are particularly important in stem cell research, where the regulation of pluripotency maintenance and lineage commitment seem to involve rapid switches between both stochastic and binary signaling events. The level of complexity, with numerous variables acting at the same time, requires multi-parametric and dynamic investigation of large numbers of single cells. This challenge may not be overcome by only using conventional bioanalytical and diagnostic approaches, therefore imaging technologies can be very supportive in this feat. Reconciliating the relationship between lineage differentiation progression and epigenetic maturation might lead to new insights into the capacities of cells derived in vitro. Likely, new technologies that combine cell monitoring and molecular analysis will be required to fully understand this relationship.

## Materials and Methods

### Stem-cell culture and endodermal differentiation

Mouse embryonic stem (ES) cells used were from the BK4 subclone of E14TG2a as previously described by Fair et al. (2005) [38]. This subclone is derived from the 129/Ola line with a deletion in the *Hprt* gene [46]. For maintenance culture, ES cells were kept on a mitotically inactivated feeder-layer of mouse embryonic fibroblasts in Dulbecco's Modified Eagle's Medium (DMEM) high glucose (Invitrogen) supplemented with 15% ES-qualified fetal bovine serum (FBS) (Atlanta Biotech), 10 µM of 2-mercaptoethanol (Sigma), 2 mM L-glutamine (Invitrogen), and 10 ng/ml of leukemia inhibitory factor (LIF) (Invitrogen). The cells were initially divided into subpopulations that were cultured either a) on 18 mm round glass cover-slips (Fisher Scientific) —that were placed into a 12-well microplate and coated with Type I collagen (Sigma)— for immunofluorescence (IF) assays, b) and in collagen-coated wells for gene-expression analysis by qRT-PCR. ES cells were removed from culture wells and seeded at a density of 8,000/cm^2^ in propagation medium (without LIF) substituted with heat-inactivated FBS and 100 ng/ml aFGF (Sigma). Cultures were allowed to grow up to seven days, then either fixed for 30 minutes in 4% paraformaldehyde (Sigma) in order to preserve the cells' three-dimensional (3-D) structure as previously described [47], [48], or harvested for RNA extraction.

### Immunofluorescence

Permeabilization to facilitate probe penetration into fixed cells and nuclei was achieved by incubation with a mixture of 0.5% saponin/0.5% triton X-100/phosphate buffered saline (PBS) followed by RNase A (100 µg/ml) treatment. The cells were blocked with 3% bovine serum albumin fraction V (BSA)/PBS before incubation with a set of two unconjugated polyclonal primary antibodies at concentration recommended by the manufacturers: primary antibodies include goat anti-Sox17, goat anti-FoxA2, goat anti-E-cadherin, rabbit anti-E-cadherin, goat anti-Oct-4, (all Santa Cruz Biotechnology, Santa Cruz, CA), rabbit anti-Sox17 (R&D Systems, Minneapolis, MN), rabbit anti-IGFR antibody (Cell Signaling Technologies, Danvers, MA) in 3% BSA/PBS overnight at 4°C. Consistently applied secondary antibodies were Alexa568-conjugated donkey anti-goat antibody (Cat. No. A-11057, Invitrogen, Carlsbad, CA) and Alexa647-conjugated chicken anti-rabbit (Cat. No. A-21443, Invitrogen), both at the concentration of 5 µg/ml in 3% BSA/PBS for one hour at 37°C. Cells were fixed for a second time in 4% paraformaldehyde/PBS for 15 min at room temperature before treating with hydrochloric acid, and blocked with 3% BSA/PBS prior to incubation with an unconjugated monoclonal mouse anti-5-methylcytosine antibody (Calbiochem, San Diego, CA), and a secondary Alexa488-linked donkey anti-mouse polyclonal IgG (Cat. No. A-21202, Invitrogen) at the concentration of 5 µg/ml, both antibodies for 1 hour at 37°C. Antibodies were diluted in blocking solution. Intermediate stringency washes after antibody incubation were performed with 0.1% BSA/0.1% Tween 20/PBS. For all other washing steps 0.1% BSA/PBS was used. The specimens were counterstained for 15 minutes at room temperature with a 1.43 µM DAPI solution (FluoroPure grade, Invitrogen), dipped in PBS, and embedded in ample mounting solution (ProLong Gold, Invitrogen) on glass slides.

### Confocal microscopy

Specimens were analyzed by confocal laser scanning microscopy using a TCS SP5 X Supercontinuum microscope (Leica Microsystems, Mannheim, Germany), equipped with a white laser: the system provides full freedom and flexibility in excitation and emission, within the continuous range of 470 to 670 nm —in 1nm increments. A coupled 405 nm diode laser line was used for excitation of DAPI fluorescence. Serial optical sections were collected at increments of 250 nm with a Plan-Apo 63×1.3 glycerol immersion lens. The pinhole size was consistently 1.0 airy unit. To avoid bleed-through, imaging of each of the four channels —MeC (488 nm), DAPI, and the two variable biomarkers (568 nm and 647 nm)— was acquired sequentially. The typical image size was 2048×2048 with a respective voxel size of 120 nm×120 nm×250 nm (x, y, and z axes), and a dynamic intensity range of 12 bits per pixel in all four channels. MeC, DAPI, and biomarker signals from optical sections were recorded into separate 3-D channels. All images were acquired under nearly identical conditions and modality settings. The drift of the settings during acquisition was considered minimal and therefore neglected.

### 3-D Image analysis and data acquisition

Image files of cells originally saved in Leica format (*.lif) were converted to a series of TIFFs using the open source ImageJ™ package. Output files were sequentially analyzed with a dedicated software we developed for high-resolution and high-content analysis —3-D Quantitative DNA Methylation Imaging (3D-qDMI) as previously decribed [32]— that contains two modules: (I) preprocessing and (II) in-depth analysis. Preprocessing entails nuclear segmentation by adaptive seeded watershed resulting in the delineation of a 3-D region of interest (ROI) for each individual nucleus. In depth analysis focuses on the extraction of MeC and DAPI features within each ROI. For the in situ characterization of cells three features were recorded for each imaged cell: (1) nuclear mean intensities of MeC, DAPI, and each marker, and (2) the intensity codistribution of MeC and DAPI, displayed as a scatter plot. The latter feature can serve as an indicator of chromatin reorganization in cells. Additionally 3D-qDMI is equipped with a fourth module, namely the statistical (homogeneity) assessment of the population based on MeC and gDNA codistributions [39].

### Quantitative real-time polymerase chain reaction

Total RNA was harvested and purified using the RNeasy Mini Kit (Qiagen, Valencia, CA). One microgram was reverse-transcribed using the QuantiTect Reverse Transcription Kit (Qiagen) following the manufacturer's directions. The resultant cDNA template was diluted 50-fold, and 1 ng of template amplified in RT^2^ SYBR Green/ROX qPCR Master Mix (SABiosciences-Qiagen) on an ABI 7300 optical thermocycler (Applied Biosystems, Foster City, CA). For each reaction, a negative control was included, in which RNase-free water (Ambion, Austin, TX) was substituted for template. Absolute levels of mRNA were normalized to β-actin, which was used as the internal control. Data (on day 7) are expressed as fold change in gene expression relative to undifferentiated embryonic stem cells (24 hps). Specific target amplification was verified by melting curve analysis. The following primer sequences were used for: β-actin, forward: 5′-ATGCTCCCCGGGCTGTAT-3′, reverse: 5′-CATAGGAGTCCTTCTGACCCATTC-3′; FoxA2, forward: 5′- AGCTACTACGCGGAGCCCG-3′, reverse: 5′-GTGTTCATGCCATTCATCCC-3′; Sox17, forward: 5′-GGCCGATGAACGCCTTT, reverse: TCTGGGTTCTGCTGTGCCA, Afp, forward: 5′- ATTGCCTCCACGTGCTGCCA-3′, reverse: 5′-GAAAATGTCGGCCATTCCCT-3′; Hex, forward: 5′-ACTACACGCACGCCCTACT-3′, reverse: 5′-CCTTTTGTGCAGAGGTCGCT-3′; IGFR, forward: 5′-GTGCCCAGGCCCGAAAGGAG-3′, reverse: 5′-GCTCCCAGGTCACCGGACCA-3′; Pax6, forward: 5′-CTGAGGAACCAGAGAAGACAGG-3′, reverse: 5′- CATGGAACCTGATGTGAAGGAGG-3′; Goosecoid, forward: 5′-TGCAAAGACGCGGTGCTCCC-3′, reverse: 5′-CCTCGTAGCCTGGGGGCGTC-3′; nestin, forward: 5′-GGAGTCAGAGCAAGTGAATG-3′, reverse: 5′-GTCTTGATCCTCGTCCCCA-3′; Nkx2.5, forward: 5′-ACCCTGACCCAGCCAAAGA-3′, reverse: 5′-GGCTTTGTCCAGCTCCACT-3′; Brachyury, forward: 5′-ATCCACCCAGACTCGCCCAATT-3′, reverse: 5′-CTCTCACGATGTGAATCCGAGG-3′; Oct-4, forward: 5′-GTTTGCCAAGCTGCTGAAGC-3′, reverse: 5′-GAAGCGACAGATGGTGGTCT-3′; and GATA-4, forward: 5′-CTGGCCAGGACTGCCGCTTC-3′, reverse: 5′-GTGCGGGAGGGCGGACTCTA-3′.
